# Targeting galectin-3 in cancer by novel and unique inhibitors of non-carbohydrate origin

**DOI:** 10.1186/s10020-025-01356-6

**Published:** 2025-09-29

**Authors:** Paulina Sindrewicz-Goral, Xiaoxin Li, Weikun Wang, Oluwatobi Adegbite, Yaoyu Pang, Thomas Gledhill, Sandra Sreenivas, Lu-Yun Lian, Lu-Gang Yu

**Affiliations:** Department of Biochemistry, Cell and Systems Biology, Institute of Systems, Molecular and Integrative Biology, University of Liverpool, Liverpool, L69 3GE UK

## Abstract

The galactoside-binding galectin-3 is a multi-mode promoter in a broad range of cancers, as well as in the pathogenesis of inflammation and fibrosis-associated diseases. It is currently a hotly pursued therapeutic target in those disease areas. Several carbohydrate-based galectin-3 inhibitors have recently demonstrated encouraging results in early phase clinical trials. This study reports the discovery of two synthetic, non-carbohydrate, small molecule compounds (named K2 an L2) as potent galectin-3 inhibitors. K2 and L2 share the same molecular composition with difference of one -NH2 group located at para (K2) or meta (L2) position at one of its aromatic rings. These novel compound inhibitors were shown to bind to galectin-3 on the canonical S-face of galectin-3 carbohydrate-recognition domain. Their binding was shown to alter galectin-3 conformation and significantly inhibit galectin-3-mediated activities in cancer cell adhesion, invasion, angiogenesis and macrophage secretion of pro-inflammatory cytokines min vitro, and markedly reduce galectin-3-mediated tumour growth and metastasis in vivo in mice as well as in chick embryos. Moreover, these novel galectin-3 inhibitors showed no detectable cytotoxicity and no genotoxicity. K2 and L2 therefore represent a unique class of novel galectin-3 inhibitors that can effectively inhibit galectin-3-mediated activities in vitro and in vivo. The discovery of these non-carbohydrate galectin-3 inhibitors offers significant promises to the development of galectin-3-targeted therapeutic drugs for the treatment of cancer and other galectin-3-mediated pathologies such as inflammation and fibrosis-associated diseases.

**Supplementary Information:**

The online version contains supplementary material available at 10.1186/s10020-025-01356-6.

## Introduction

Galectin-3 is a member of the galactoside-binding galectin family. It is expressed by many types of human cells, particularly epithelial and immune cells, and plays multifaceted roles in cell–cell and cell-environment communications (Newlaczyl and Yu 2011; Girotti et al. 2020).

Overexpression of galectin-3 is a common feature in cancers of all major types such as breast, prostate, colorectal and lung cancers (Newlaczyl and Yu 2011). Higher galectin-3 expression in cancer and in circulation is associated often with tumour aggressiveness and poorer patient outcomes. More and more evidence has shown that galectin-3 is a multi-functional, multi-mode promoter in cancer development, progression, metastasis and immune suppression (Newlaczyl and Yu 2011; Girotti et al. 2020) in various stages such as in tumour cell adhesion, invasion, angiogenesis and immune evasion. The multi-mode galectin-3 actions in cancer promotion is known to derive largely from its versatile ability to interact with various galactose-terminated glycans carried on many cell surface and basement matrix molecules such as growth factor receptors, death receptors, adhesion molecules and immune check-point molecules (Barrow et al. 2011a; Marino et al. 2023; Liu and Rabinovich 2005). For example, binding of galectin-3 to integrins or laminin enhances tumour cell adhesion and subsequent invasion into the surrounding tissues at the primary tumour sites (Yang et al. 2017). Binding to cell surface mucin protein MUC1 increases circulating tumour cell heterotypic adhesion to the blood vascular endothelium (Zhao et al. 2009), and promotes tumour cell–cell homotypic aggregation for the formation of circulating tumour emboli (Zhao et al. 2010) that facilitate tumour cell survival, extravasation and colonization at distant sites. Binding of galectin-3 to checkpoint LAG3 and CTLA4 on immune cell surface prevents immune cell activation and penetration thus to aid tumour cell evasion from immune surveillance (Kouo et al. 2015). Binding of galectin-3 to VEGFR enhances VEGFR activity and promotes new blood vessels formation and metastasis (Markowska et al. 2010). Binding of galectin-3 to cell surface glycans also promotes the formation of an inflammatory and fibrotic environment within the tumour (Bouffette et al. 2023) that supports tumour growth and resistance to cancer therapeutic treatments including chemo-, radio-, and immune-therapies (Navarro et al. 2020).

Due to its broad involvement in promotion of almost all cancer hallmarks (Girotti et al. 2020) and its profound impact on cancer progression, metastasis and immune modulation, galectin-3 is now widely regarded as a very promising therapeutic target for cancer treatment (Liu and Rabinovich 2005; Ahmed et al. 2023; Dings et al. 2018). Several galectin-3 targeted therapeutic strategies, all aimed to prevent galectin-3 binding to its ligands, have been explored in the past few years by many research laboratories and biotech companies. These approaches include the development of galectin-3 inhibitors from natural polysaccharides, peptide mimics, monoclonal antibodies, glycomimetic and carbohydrate-compound conjugates (Marino et al. 2023; Ahmed et al. 2023; Sethi et al. 2021). Some of those inhibitors have shown promising results in early phase clinical trials (Marino et al. 2023; Aslanis et al. 2023; 2023).

We report in this study the discovery of two non-carbohydrate small molecule compounds as potent galectin-3 inhibitors. These purely synthetic galectin-3 compound inhibitors are shown to effectively inhibit galectin-3 binding to its ligands, galectin-3-mediated cancer cell activities in vitro and significantly reduce tumour growth and metastasis in vivo in chick embryos and in mice.

## Materials and methods

### Materials

Monoclonal anti-galectin-3 antibody, ELISA kits for IL-6, TNFα and IL-1β, were obtained from R&D Systems (Abingdon, UK). Luciferase Assay Reagent (LAR) and Passive Lysis Buffer (PLB) were obtained from Promega (Southampton, UK). FAST OPD tablets and asialo fetuin (ASF) were obtained from Sigma-Aldrich (Dorset, UK). Falcon 8 µm pore trans-well inserts, Vybrant™ DiI fluorescent Cell-labelling solution, LDH cytotoxicity kits were from Thermo Fisher Scientific (Waltham, USA). Matrigel Matrix was purchased from BD Biosciences (Wokingham, UK). EBM-2 Bullet kits were obtained from Lonza (Verviers, Belgium). Human recombinant galectin-3 was expressed in *E.Coli* and purified as previously described (Sindrewicz et al. 2019a). CRISPR/Cas9 galectin-3 knockout plasmid (sc-417680), galectin-3 HDR plasmid (sc-417680-HDR) and Plasmid Transfection Medium (sc-108062) were obtained from Santa Cruz Biotechnology (Santa Crus, CA).

### Cells

Human colon cancer SW620 cells were obtained from European Collection of Cell Cultures (Salisbury, UK), the human breast cancer MDA-MB-231 and pancreatic cancer PANC-1 cells were kindly provided by Professor David Fernig and Professor Eithne Costello-Goldring (University of Liverpool) respectively. The cells were cultured in Dulbecco’s modified Eagle medium (DMEM) containing 10% fatal calf serum. Human umbilical vein endothelial cells (HUVECs) were obtained from Lonza (Verviers, Belgium) and cultured in EBM-2.

THP-1 monocytes were kindly provided by Dr Jack Zhang (University of Liverpool). THP-1 (5 × 10^5^ cells/ml) were incubated with 10 ng/ml Phorbol 12-myristate-13-acetate (PMA) for 48 h at 37℃ for differentiation of the monocytes to macrophages as previously described (Wan et al. 2023).

### Galectin-3 binding to its ligand

Ninety-six well plates were coated overnight with 20 µg/ml asialo-fetuin (ASF) in coating buffer (15 mM Na_2_CO_3_ 17 mM NaHCO_3_ pH 9.6). The wells were washed twice with PBS and incubated with blocking buffer (1% BSA in PBS) for 1 h. The supernatants were removed and replaced with 5 µg/ml human recombinant galectin-3 without or with different concentrations of compound inhibitors in blocking buffer for 1 h at room temperature. The wells were washed and incubated with 1 μg/ml anti-galectin-3 antibody for 2 h. Following addition of peroxidase-conjugated secondary antibody for 1 h, FAST OPD development solution was added for 15–20 min. The reaction was terminated by addition of 4 M sulphuric acid. The plates were read at 492 nm with reference wavelength at 595 nm by a microplate reader (Tecan, Switzerland).

### Analysis of compound-galectin-3 binding affinity

The binding affinity of the inhibitors to galectin-3 was determined by Intrinsic Tryptophan Fluorescence Spectroscopy (TFS) (Sindrewicz et al. 2019a). The Galectin-3 concentration used was 10 µM and the titration concentrations of the inhibitors were from 0 to 224 µM. DMSO/control buffer (PBS with 2 mM EDTA) titrations were also included and used for background subtraction.

### Analysis of galectin-3-compound interaction by molecular docking

Molecular docking analysis was performed using three programmes -Autodock VinaVersion 1.1.2, Boltz-2 and DynamicBind Version 2. The latter two programmes were access through the Neurosnap platform of Bioinformatics Tools (https://neurosnap.ai/). Of the three methods, DynamicBind yielded the most consistent results and the protocol for this method will be discussed here. The crystal structure of galectin-3 3T1L (with the bound ligands and water molecules removed) was used as a template. The inputs were the PDB coordinates for galectin-3 and the SMILES formulae for K2 and L2. For each complex, the recommended default parameters were used: 20 models were predicted, the inference steps set to 20, and both the post-prediction relaxation and noise in the final step of the reverse diffusion carried out. Following molecular docking, potential galectin-3-compound interactions were identified using Protein–Ligand Interaction Profiler (PLIP) (Adasme et al. 2021). Pymol was used for the molecular visualization and analysis.

### Galectin-3 knockout by CRISPR/Cas9

MDA-MB-231 and PANC-1 cells were seeded into 6-well plate (2 × 10^5^ cells) and cultured at 37^0^C for 24 h. Galectin-3 CRISPR/Cas9 Knockout plasmid (1 µg) and galectin-3 HDR plasmid (1 µg) was mixed with Plasmid Transfection Medium (25 µl) for 5 min before addition to a mixture of lipofectamine STEM Transfection Reagent (STEM00001) (1 µl) and Plasmid Transfection Medium (25 µl) for 10 min at room temperature. The cells in the 6-well plates were washed and introduced with the Plasmid DNA/Lipofectamine STEM Transfection Reagent complex at 37^0^C for 24 h. The culture medium was replaced with fresh antibiotics-free medium for further 24 h incubation before introduction of 10 µg/mL puromycin-containing medium every three days. Successful co-transfection of the CRISPR/Cas9 KO Plasmid and HDR Plasmid was confirmed by expression of red fluorescence protein of the cells. Galectin-3 knockout in the cells was further confirmed by immunoblotting.

### Galectin-3 knockdown by shRNA and transduction with luciferase

Galectin-3 knockdown in SW620 cells with control and galectin-3 shRNA were described previously (Duckworth et al. 2015). The transfected cells of control SW620 and galectin-3-knockdown SW620Gal3KD cells were further transduced with lentiviral pHIV-Luc-ZsGreen plasmid (Addgene plasmid #39196) (Taylor et al. 2018). Both SW620 and SW620Gal3KD cells were seeded into 24-well plate (1 × 10^4^ cells/well) in DMEM 10% FBS and incubated for 4–5 h before treatment with the pHIV-Luc-ZsGreen plasmid at 1:5 Multiplicity of Infection (MOI for four days at 37^0^C 5% CO_2_.The positively transduced cells were selected by FACS using FACSAria III Cell Sorter (BD Biosciences, UK).

### Cancer cell adhesion to matrix proteins

Ninety-six well plates were coated with 50 µl Matrix (1:30 dilution in PBS) at 37 °C for 1 h. Cancer cells were labelled with fluorescent cell-labelling solution DiI at 37 °C for 20 min and were resuspended into 3 × 10^4^cells/ml in serum-free medium. The matrix-coated 96-well plates were washed twice with PBS before introduction of 100µ cell suspension with different concentrations of inhibitors or PBS/DMSO (control) for 1 h at 37℃. The plates were washed twice with PBS before 5 ~ 10 randomly selected Field of Views (FOVs) were recorded by fluorescence microscope and number of adhesion cells were quantified by Image J.

### Cancer cell adhesion to laminin

White wall 96-well plates were coated with laminin (20 µg/ml) in coating buffer (15 mM Na2CO3 17 mM NaHCO3 pH 9.6) overnight at room temperature. ZsGreen- and luciferase- transfected SW620, SW620Gal3KD cells (5 × 10^5^ cells/ml) were pre-incubated without or with 10 µM inhibitors for 10 min before added into laminin-coated plates for 30 min at 37^0^C. The plates were washed twice with PBS before lysed with PLB for 5 min on a shaker. Luciferase Assay Reagent was added, and luminescence was measured using Tecan Infinite F200 microplate reader (Tecan, Switzerland).

### Cancer cell adhesion to vascular endothelium

Effect of the compound inhibitors on galectin-3-mediated cancer cell adhesion to vascular endothelium was investigated using human umbilical vein endothelial cells (HUVECs). HUVECs (1.5 × 10^5^ cells/ml) were seeded into 96-well plates in complete EBM-2 medium and incubated at 37^0^C 5% CO_2_ for 48–72 h until a monolayer was formed. ZsGreen- and luciferase- transfected SW620, SW620Gal3KD cells (5 × 10^5^ cells/ml) were added to the HUVEC monolayer without or with 10 µM inhibitor for 30 min at 37^0^C. The cells were washed twice with PBS before lysed with PLB. The cell lysates were transferred into new wells of white-walled 96-well plates, LAR was added and luminescence was measured using Infinite F200 Microplate reader.

### Cancer cell invasion though vascular endothelium

HUVECs were plated at a density of 1.5 × 10^4^ cells in 8 µm pore transwells in 24-well plate and cultured for three days to allow formation of a tight monolayer. The integrity of HUVEC monolayer was confirmed by measuring trans-endothelial electric resistance (TEER) and only monolayers with TEER reading > 800 Ω/cm^2^ were used for the invasion assay. The HUVEC monolayers were washed with PBS before introduction of ZsGreen- and luciferase-transfected SW620, SW620Gal3KD cells (5 × 10^5^cells/ml) in serum-free DMEM with 0.5 mg/ml BSA, without or with 10 µM inhibitor. The cells were incubated at 37^0^C 5% CO_2_ for 16–18 h. The cells at the upper side of the trans-well inserts were gently removed with cotton swabs and the inserts were washed twice with PBS. The cells that migrated to the bottom side of the trans-wells were lysed with PLB buffer for 10 min. The lyse was introduced with LAR for 5 min before luminescence was measured using Infinite F200 Microplate reader.

### Angiogenesis (vascular tubule formation) measurement

Matrix (50 µl) was added to 96-well plates for 60 min at 37^0^ C to allow gel formation. HUVEC cells (1 × 10^5^cells/ml in 1:1 EGM/EBM2 media) was introduced to the plates without or with galectin-3 (2 µg/ml) or different concentrations of compound inhibitors for 24 h at 37 °C. Endothelial cell tube formation was examined with Leica DMLA Laser Microscope. The total number of branching points and the lengths of tubes were calculated as previous described (Duckworth et al. 2015).

### Macrophage pro-inflammatory cytokine secretion

Effect of the compound inhibitors on macrophage secretion of pro-inflammatory cytokines were assessed in THP-1 differentiated macrophages. THP-1 cells were differentiated into macrophages by incubation with 10 ng/ml PMA for 48 h at 37℃ before incubation with fresh medium without or with 10 µg/ml galectin-3 and various concentrations of compound inhibitors for 24 h at 37℃ (Wan et al. 2023). The culture medium was collected and the concentrations of IL-6, TNFα and IL-1β in the culture medium were determined by IL-6, TNFα and IL-1β ELISA.

### Cytotoxicity

SW620 cells (5 × 10^4^ cells/ml) were seeded into 96-well plate in DMEM and incubated at 37^0^C 5% CO_2_ for 24 h. HUVECs (1.0 × 10^5^ cells/ml) was seeded in EBM medium and incubated for 24–36 h until monolayer formation. Compound inhibitors at different concentrations were then introduced to the cells (10 × lysis buffer was included as maximum cytotoxicity control) for 24 h. The culture medium was transferred to a new plate and was mixed with equal volume of reaction mixture provided in the Lactate dehydrogenase (LDH) cytotoxicity kit for 30 min at room temperature before the absorbance was measured at 490 nm (with reference at 680 nm) by a spectrophotometer.

### Genotoxicity

Effect of the compound inhibitors on genotoxicity was determined using two *Salmonella typhimurium* strains of TA 98 and TA 100 in the presence and absence of metabolic activation (S9) by the Xenometric Ames test kit (Xenometrix, Switzerland). Each test included the vehicle and positive controls. Briefly, the compounds were serially diluted in 24-well plates with or without the presence metabolic activation system (S9) and was incubated for 90 min at 37 °C in 250 rpm in an orbital shaking incubator. TA98 and TA100 were then introduced to the wells for 90 min. Fifty µL aliquots from each well was dispensed into 384 well plates and incubated for 48 h at 37˚C in an incubator before positive wells were scored.

### Circular Dichroism (CD) spectroscopy analysis

JASCO J-1500 spectropolarimeter equipped with a Peltier temperature control system was used to analyse galectin-3 secondary structures in response to Ligand binding. All measurements were conducted in a 1mm pathlength quartz cuvette. Galectin-3 (15 µM) and ligands (20 µM) were prepared in 10 mM sodium phosphate buffer (Na₂HPO₄ pH 7.4), following buffer exchange to ensure uniform sample conditions. Spectra were recorded at far-UV region from 260 to 195 nm at 20 °C. Baseline correction was performed using the corresponding buffer spectra.

### Tumour growth and metastasis in chicken embryos

Ethical approval for the experiments involving chick embryos up to embryonic day 14 was obtained from the Liverpool Animal Welfare and Ethical Review Body.

Fertilised white leghorn chicken eggs were obtained from Lees Lane Poultry (Wirral, UK). Eggs, incubated at 37^0^C at 35–40% humidity, were windowed on embryonic day 3 (E3). After 4 ml albumin was removed, the window was sealed using adhesive tape and the eggs were returned into 37^0^C incubator until embryonic day 7. GFP- and luciferase-expressing SW620 or SW620Gal3KD cells at 2.5 × 10^6^ cells in 8 µl of medium were embedded on the chorioallantoic membrane (CAM). K2 (24 µM) was injected into the allantoic cavity on the following day and the tumour formation and growth was recorded by fluorescence microscopy and IVIS imaging on embryonic day 14. Immediately following tumour analysis on day 14, the embryos were decapitated and dissected and metastasis of tumour cells in organs was examined under fluorescence microscopy.

### Tumour growth and metastasis in mice

Female Balb/c nude mice aged 6–7 weeks were purchased from Charles River Laboratories (Margate, UK) and maintained in specific pathogen-free conditions with a 12:12 h light:dark cycle. All animal studies were conducted with UK Home Office and local ethics committee approval.

In the tumour growth study, 18 Bob/c nude mice were grafted with 1 × 10^6^ luciferase-transfected SW620 cells subcutaneously. The mice were randomly divided into two equal groups (9 mice/group). K2 (5 mg/kg) or PBS (control) was injected i.p. 3 times a week for 4 weeks starting from day one. Tumour growth were monitored by clipers twice a week and also by IVIS imaging weekly.

In the metastasis study, 32 Balb/c female nude mice were randomly divided into four equal groups (8 mice/group). ZsGreen- and luciferase-transfected SW620 and SW620Gal3KD cells (1 × 10^6^ cells) were intravenously injected into mice via the tail vein. K2 (10 mg/kg/day) or PBS (control group) was intravenously administrated into mice for 5 consecutive days in the first week then subcutaneously once a week for seven weeks. Metastasis was monitored by IVIS imaging weekly. The animals were killed, and Lungs were excised and the number of metastasis nodules on the surface of the animal Lungs were quantified under a dissection microscope. Lung weight was also measured as an indicator of tumour burden. The lungs were fixed overnight in 4% formalin, processed and paraffin-embedded. Sections were stained by Hematoxylin and Eosin for examination of tumour formation inside lungs.

### Statistical analysis

One-way analysis of variance (ANOVA) followed by Bonferroni correction or Dunnett's Test was used for multiple comparisons, and two tailed independent t-test was used for comparison of two groups using SPSS or R. Difference was considered significance when *p* < 0.05.

## Results

### Identification of two non-carbohydrate compounds as potent galectin-3 inhibitors

**Fig. 1:**
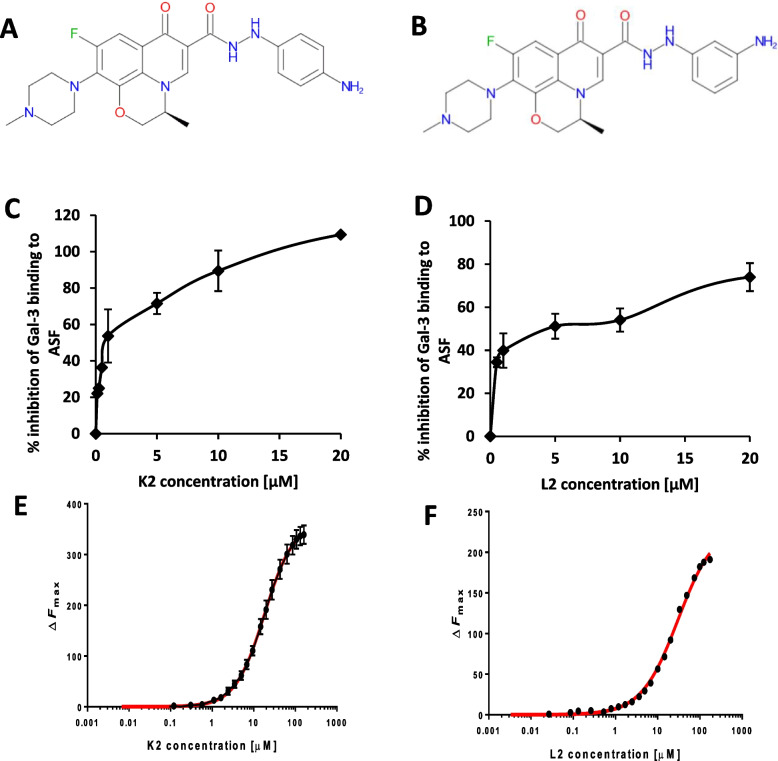
K2 and L2 inhibit galectin-3 binding to its ligand. The chemical structures of the compounds are shown in **A** (K2) and **B** (L2). Inhibition of K2 (**C**) and L2 (**D**) on galectin-3 (5 µg/ml) binding to asialo-fetuin (ASF, 20 µg/ml) was determined by galectin-3 ELISA. K2 and L2 both cause dose-dependent inhibition of galectin-3 binding to ASF. The binding affinity of K2 (**E**) and L2 (**F**) to galectin-3 was determined by TFS. The data are shown as mean ± SD of triplicate assessments

Screening a few compound libraries including that from Redx Oncology Ltd UK (with a total > 1000 compounds) by competitive galectin-3 ELISA (with asialo fetuin as the binding ligand) identified two small molecule compounds from the Redx Oncology library, named K2 and L2, as potent galectin-3 binding inhibitors. K2 and L2 have the same molecular composition (M_W_ = 466 Da) with difference of one -NH_2_ group located at para (K2) or meta (L2) position at one of its aromatic rings (Fig. 1A and B). K2 and L2 both showed inhibition of galectin-3 binding to its ligand asialo-fetuin in a dose-dependent manner with IC_50_ at approximately 1 and 5 µM for K2 and L2, respectively (Fig. 1C and D). Analyses of K2 and L2 binding to galectin-3 using tryptophan fluorescence spectroscopy (TFS) (Sindrewicz et al. 2019) showed binding affinities of 18.1 ± 1.5 µM and 30.2 ± 1.4 µM, respectively for K2 and L2 (Fig. 1E and F).

### Molecular docking predicts binding of K2 and L2 to the canonical galectin-3 CRD

**Fig. 2:**
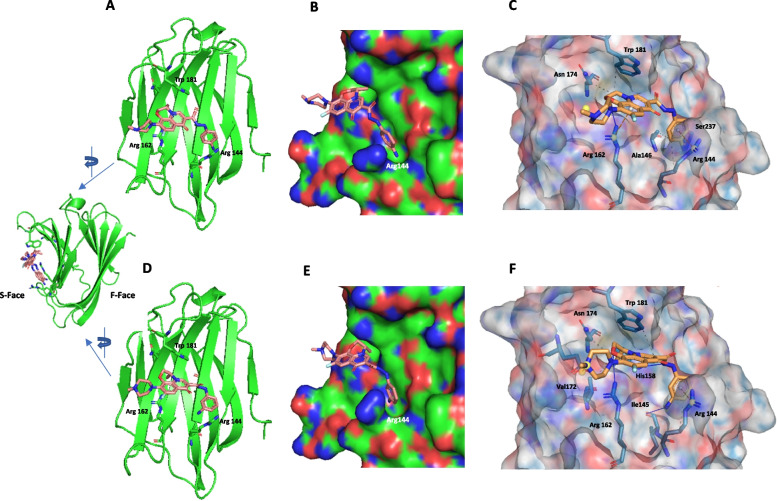
Molecular docking analysis predicts binding sites of K2 and L2 to the S-face of galectin-3 CRD. The predicted binding sites of K2 (**A**-**C**) and L2 (**D**-**F**) on galectin-3. **A**, **D** K2 and L2 bind to the S-face of galectin-3 CRD. K2 and L2 are represented as orange sticks, Galectin-3 in green cartoon, and the side chains of Arg144, Arg162 and Trp181 are highlighted. The S- and F-faces of Galectin-3 are indicated in the inset. **B**, **E** Expanded view of binding pocket showing the absence of interactions between the piperazine moiety and the protein. Note the pockets formed by Arg144 guanidino side-chain into which the anilino group in located. **C**, **F** DynamicBind modelled structure of compound K2 (**C**) and L2 (**F**) complexed to galectin-3 (PDB 3T1L). Colour key: Protein – Light Grey surface, Amino acids: Light Blue – Carbon, Blue – Nitrogen, Red – Oxygen. Ligand: Orange – Carbon, Blue – Nitrogen, Red – Oxygen, Light cyan – Fluorine. Interactions between Ligand and Protein: White balls—Aromatic centroids; Red dashed line—π-cations interactions; Blue solid lines – Hydrogen bonding; Grey dashed Lines – hydrophobic interactions. Figure was created in PyMOL Molecular Graphics System, Version 1.7.4.4, Schrödinger, LLC

Both K2 and L2 were docked onto galectin-3 using three programmes -DynamicBind (Lu et al. 2024), Boltz-2 (Passaro et al. 2025) and Autodock Vina (Eberhardt et al. 2021). Boltz-2 and DynamicBind are cutting-edge generative learning algorithms designed for small-molecule docking. We tested the three methods using our own experimentally-determined, but yet published crystal structures of protein–ligand complexes; this removed any biasness as the data sets had not been used for machine training by any of the programmes. In all three methods, the ligands docked onto the S-face of the consensus sugar binding site of galectin-3 CRD (Fig. 2A and D), although with varying degrees of consistency. DynamicBind had the highest success rate with all iterations for each ligand giving very similar results. This was followed by Boltz-2 although there were more outliers than the results from DynamicBind. Targeted AutoDock Vina returned the most diverse set of results with the ligand adopting many different poses over a wider region of the protein surface. The results from DynamicBind docking are discussed here. The intermolecular interaction between residues on galectin-3 and atoms on K2 and L2 are summarised in the Table S1, with the binding affinities and LDDT (confidence) scores for the top 10 poses summarised in Table S2 (for K2) and Table S3 (for L2).

In both the K2-galectin-3 and L2-galectin-3 complexes, the piperazine moiety does not appear to make contacts with the protein (Fig. 2B and E), with the aromatic atoms of both the tricyclic-fluoro quinolone core and carbohydrazide anilino side-chain forming the main interactions with protein residues (Fig. 2C and F). In both the K2 and L2 complexes, pi-cation interactions from Arg 144 guanidino side-chain to the anilino group are present, with K2 showing an extra set of pi-cation interactions from Arg 162 to the tricyclic-fluoro quinolone core (Table S1). The role of Arg144 is interesting; in both complexes, its side-chain adopts a conformation to provide a pocket for the pi-cation interactions to occur (Fig. 2B and E). The pi-stacking interactions involving Arg144 has been found to be responsible for conferring high ligand-binding affinities in other campaigns to develop galectin-3 inhibitors (Bouffette et al. 2023). Arg 144 is not a conserved residue amongst most galectins whereas Arg 162 is more conserved. Arg 186, a conserved residue in galectins, is also located on the S-face and had previously been shown to form pi-stacking interactions in some galectin-3 inhibitors (Zetterberg et al. 2022); however, this protein residue is not involved with interactions with either K2 or L2. Hence, the differences between K2/L2 and other inhibitor interactions, and that the non-conserved Arg144 forms the main pi-cation interactions in the K2/L2 complexes, hence contributing to improving affinities, demonstrate these compounds can be selective inhibitors of galectin-3.

K2 makes hydrophobic contacts with Arg144, Ala146, Asn 174 and Trp 181, and hydrogen bonds with Arg162, Ser 237. L2 makes hydrophobic interactions with Arg144, Val172, Asn 174 and Trp181, and hydrogen bonds with Ile14, His 158 and Arg162. Most of the hydrophobic contacts and hydrogen bonds are with conserved residues in galectins.

The only difference between K2 and L2 is the position of the amino group on the anilino side chain (Fig. 1) which is in, respectively, position 4 and 3 in K2 and L2 (Table S1); the nitrogen of K2 hydrogen bonds with Arg162 side chain whereas the nitrogen of L2 hydrogen bonds with Ile145 main chain and these amino acid residues are conserved amongst the galectins. Hence, it is unlikely that these amino groups would not confer selectivity for the different galectins.

### K2 and L2 inhibit galectin-3-mediated cancer cell adhesion to basement proteins

Galectin-3 is known to promote tumour cell adhesion to and invasion through basement matrix in tumour cell breakout at the primary tumour site by interaction with a number of basement matrix glycoproteins such as laminin (Nangia-Makker et al. 2008). Having shown inhibition of the compounds on galectin-3 binding to its ligand, effect of these compound on galectin-3-mediated tumour cell adhesion to matrix proteins was assessed.

**Fig. 3:**
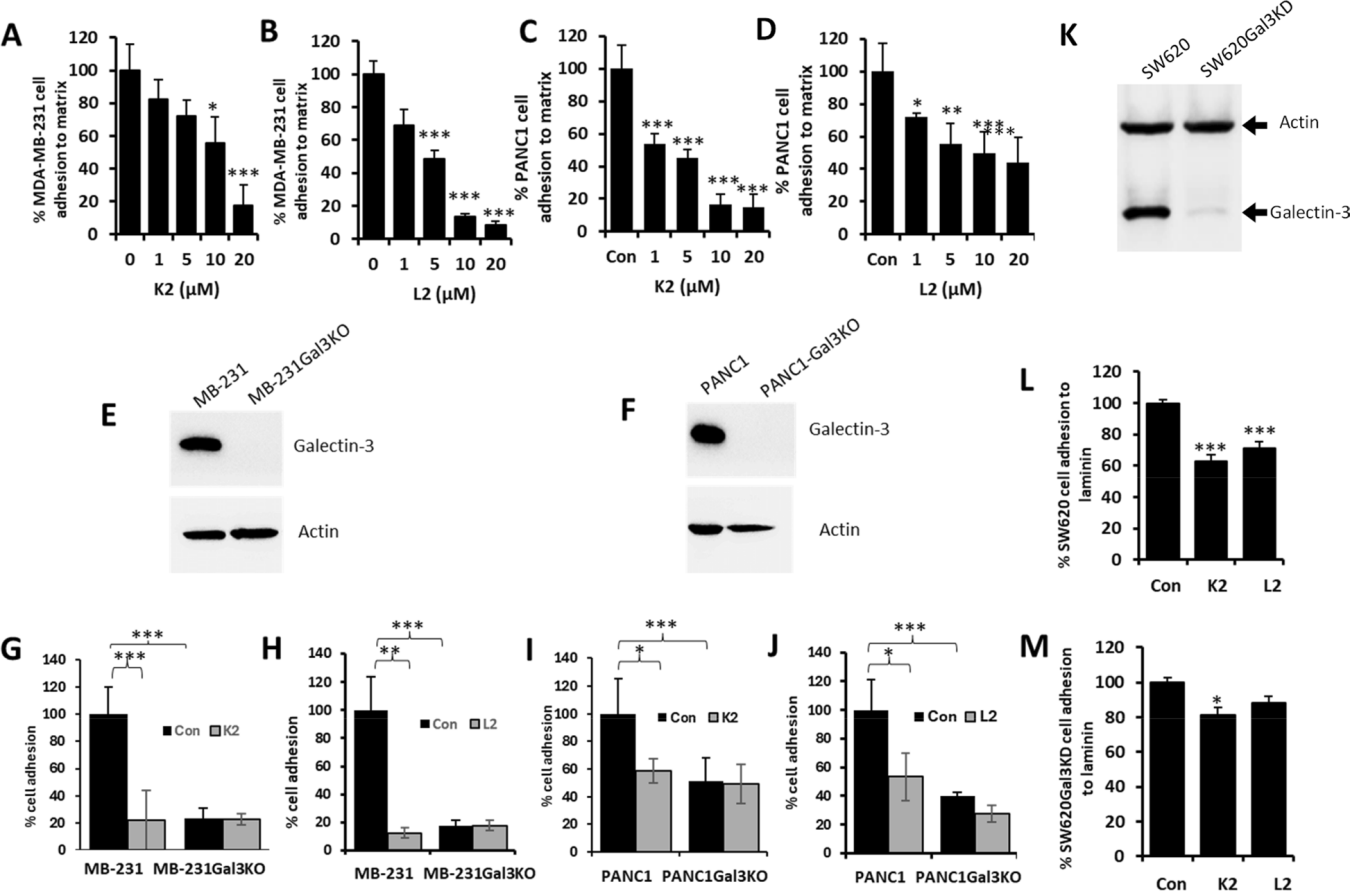
K2 and L2 inhibit galectin-3-mediated cancer cell adhesion to matrix proteins. Adhesion of human breast cancer MDA-MB-231 (**A**, **B**) and pancreatic cancer PANC1 (**C**, **D**) cells to basement matrix was determined in the absence or presence of various concentrations of K2 (**A**, **C**) and L2 (**B**, **D**). K2 and L2 both dose-dependently inhibit MDA-MB-231 and pancreatic PANC1 cell adhesion to the basement matrix. Galectin-3 expression in MDA-MB-231 (**E**) and PANC1 (**F**) cells without and with galectin-3 knockout by CRISPR/Cas9 was assessed by galectin-3 immunoblotting. The blots were striped and reprobed with anti-actin antibody for verification of protein loading. K2 (**G**, **I**) and L2 (**H**, **J**) at 10 µM inhibit MDA-MB cell adhesion but not galectin-3 knockout MDA-MB-231Gal3KO (**G**, **H**) and PANC1Gal3KO (**I**, **J**) cells. Expressions of galectin-3 in SW620 and galectin-3 shRNA knockdown cells (SW620Gal3KD) was assessed by galectin-3 and actin dual blotting (**K**). Adhesion of SW620 (**L**) and SW620Gal3KD (**M**) cells to laminin was assessed in the absence or presence of 10 µM K2 or L2. K2 and L2 inhibit adhesion to laminin of SW620 cells but much less so of SW620Gal3KD cells (**M**). The data are shown as mean ± SD of three to four independent experiments, each in triplicate. *p < 0.05, **p < 0.01, ***p < 0.001 (ANOVA and post-hoc)

The presence of both K2 and L2 was shown to cause significant inhibition of adhesion to basement matrix of human breast cancer MDA-MB-231 (Fig. 3A, B), pancreatic cancer PANC-1 (Fig. 3C, D) and colon cancer SW620 cells (Fig. 3L). To determine whether this inhibition of the compounds on tumour cell adhesion was related to inhibition of the galectin-3-mediated actions, expression of galectin-3 in these cells were either knocked out by CRISPR/Cas9 (MDA-MB-231 and PANC-1) or knock-down (SW620) by shRNA. CRISPR/Cas9 galectin-3 transfection completely knocked out the expression of galectin-3 in MDA-MB-231 and PANC1 cells (Fig. 3E, F). ShRNA transfection led to 85% reduction of galectin-3 expression in SW620 cells (Fig. 3K). It was found that K2 and L2 completely lost their inhibitory effects on adhesion of MDA-MB-231 and PANC1 cells when galectin-3 was knocked out in these cells (Fig. 3G-J). K2/L2-mediated inhibition of SW620 cell adhesion to laminin (Fig. 3L) was also significantly reduced when galectin-3 expression in the cells was knocked down by shRNA (Fig. 3M). Together, these results indicate that K2 and L2 can significantly inhibit galectin-3-mediated tumour cell adhesion.

### K2 and L2 inhibit galectin-3-mediated cancer cell adhesion to and invasion through vascular endothelium and angiogenesis

Adhesion to and invasion through the blood vascular endothelium are two critical steps in tumour cell spreading in metastasis. Extracellular/circulating galectin-3 is known to be critically involved in these processes (Zhao et al. 2009; Glinsky et al. 2001) as well as in promotion of angiogenesis (Markowska et al. 2010; Funasaka et al. 2014). It was found that K2 and L2 both significantly inhibited SW620 cell adhesion to (Fig S1A) and invasion through (Fig S1B) human umbilical vein endothelial cells. This effect of the compounds on SW620 cell invasion was completely lost when galectin-3 expression in the cells was knocked down by shRNA (Fig S1C). The presence of either K2 or L2 was shown to also cause dose-dependent inhibition of galectin-3-mediated vascular tubule formation (Fig S1D, E). These suggest that K2 and L2 are capable to effectively inhibit a range of galectin-3-mediated activities in cancer progression and metastasis.

### K2 inhibits galectin-3-mediated secretion of pro-inflammatory cytokines from macrophages

**Fig. 4:**
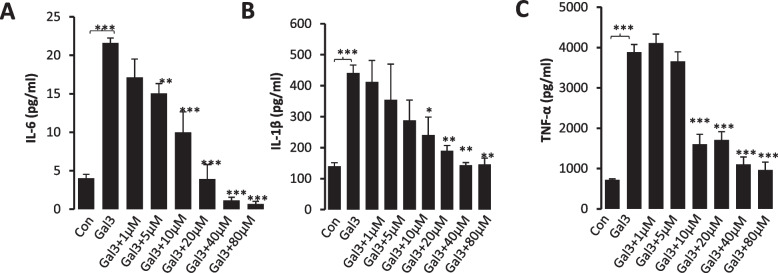
K2 inhibits galectin-3-induced secretion of pro-inflammatory cytokines from macrophages. THP-1-differentiated macrophages were treated with galectin-3 in the absence or presence of different concentrations of K2 for 24 h. The levels of IL-6 (**A**), IL-1β (**B**) and TNFα (**C**) in the culture media were analysed by cytokine ELISA. Galectin-3 markedly increased secretion of IL-6, TNFα and IL-1β from macrophages and the presence of K2 caused dose-dependent inhibition of galectin-3-induced secretion of each of these pro-inflammatory cytokines. The data are shown as mean ± SD of three independent experiments. *p < 0.05, **p < 0.01, *** *P* < 0.001 (ANOVA and post-hoc)

Excess secretion of pro-inflammatory cytokines (e.g. IL-6, IL-1β, TGFα) by immune cells is critically involved in tumour cell avoidance of immune surveillance in the tumour microenvironment as well as in inflammation (Lan et al. 2021). Galectin-3 is known to be a promoter of the secretion of pro-inflammatory cytokines such as IL-6 from endothelial (Chen et al. 2014), fibroblasts (Filer et al. 2009) and immune cells (Chen et al. 2015). It was found here that the presence of galectin-3 at 10 µg/ml, a galectin-3 concentration that is close to that found in patients with metastasis colorectal cancer (Barrow et al. 2011b), led to marked increase of the secretion of IL-6 (> fivefold), IL-1 (> threefold) and TNFα (> threefold) from THP-1 differentiated macrophages (Fig. 4A-C). The presence of K2 caused a dose-dependent inhibition of the secretion of these pro-inflammatory cytokines from macrophages. This indicates a powerful effect of these compounds on inhibition of galectin-3-mediated secretion of pro-inflammatory cytokines from immune cells.

### K2 and L2 have low cytotoxicity and genotoxicity

**Fig. 5:**
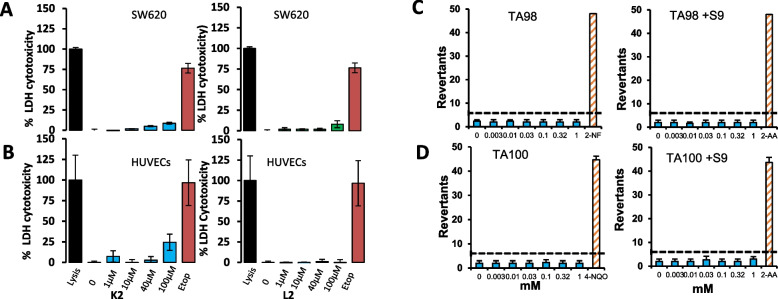
K2 and K2 show no detectable cytotoxicity and genotoxicity. K2 and L2 cytotoxicity in human colon cancer SW620 (**A**) and vascular endothelial HUVECs (**B**) was assessed in the absence or presence of various concentrations of K2 and L2 (100 µM Etoposide, Etop, was included as positive control and cell lysis by lysis buffer as maximum LDH activity) by LDH release assay. The data was expressed as percentage ± SD to max LDH activity caused by cell lysis. K2 and L2 show no detectable cytotoxicity to human HUVECs and SW620 cells at < 100 µM while Etoposide showed almost 100% cytotoxicity to the cells in the same condition. C and D: Mutagenic potential of K2 was assessed in two *Salmonella typhimurium* strains TA98 and TA100 without and with metabolic activation S9 by the Xenometric Ames test kit. K2 showed no mutagenic activity (up to 1 mM tested) in either TA98 (**C**) or TA100 (**D**) salmonella strains without or with metabolic activation [note, the positive controls 2NF (2-nitrofluorene), 2AA (2-amibnoanthacene) and 4-NQQ (4-NitroquinolineN-oxide) each produced a distinct increase of revertant colony count in the test]. *p < 0.05, **p < 0.01, ***p < 0.001 (ANOVA and post-hoc)

The cytotoxicity of K2 and L2 were investigated in both normal human vascular endothelial HUVEC cells and human colon cancer SW620 cells. K2 and L2 showed almost no detectable effect on LDH release from both SW620 (Fig. 5A) and HUVEC (Fig. 5B) cells at up to 100 µM tested concentration while in contrast, the chemotherapeutic drug Etoposide showed > 70% cytotoxic to SW620 and almost 100% to HUVECs.

When mutagenesis potential was assessed for K2 in two *Salmonella typhimurium* strains of TA98 and TA100, no detectable mutagenic activity was seen at up to the 1 mM tested concentrations in either TA98 (Fig. 5C) or TA100 (Fig. 5D) strains without or with metabolic activation by S9. These results indicate these compounds inhibitors have very low cytotoxicity and no genotoxicity.

### Binding of K2 and L2 to galectin-3 induces galectin-3 conformation changes

**Fig. 6:**
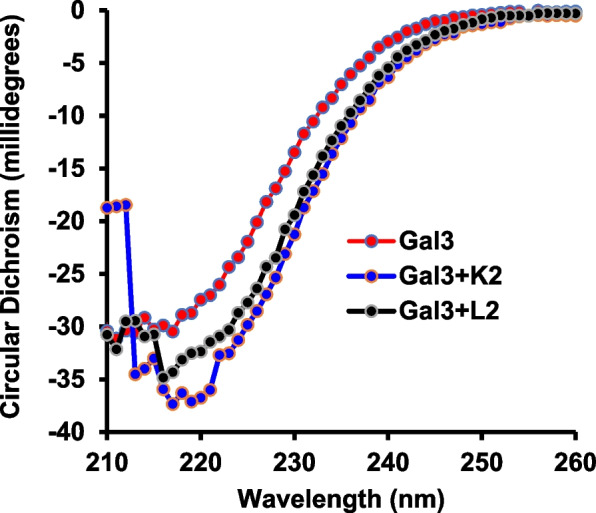
Binding of the compounds induces galectin-3 conformation changes. Interaction of K2/L2 with galectin-3 was analysed by CD spectroscopy. All samples were matched with corresponding DMSO concentrations to ensure consistent baselines. Binding of K2 and L2 to galectin-3 induces galectin-3 conformation changes

To gain further insight into the interaction of K2 and L2 with galectin-3 and its influence on galectin-3 actions, interaction of the compounds with galectin-3 in solution was analysed by Circular Dichroism spectroscopy. CD spectroscopy conducted in the far-UV region is a highly sensitive way to detect changes of protein secondary structure in solution (Duckworth et al. 2015) and is often used to provide unequivocal evidence of conformation changes of a protein upon ligand binding. The presence of K2 or L2 both caused distinct changes of galectin-3 secondary structure above 215 nm (Fig. 6, Fig S2). The range of 216-220 nm captures relevant structural changes of proteins, such as galectin-3, that is rich in β-sheet content, for which characteristic signals remain observable and interpretable around 216–220 nm. The observed decrease in ellipticity at 218 nm of galectin-3 in response to K2 and L2 is a characteristic β-sheet structure alteration (Greenfield 2006), indicates conformation changes of galectin-3 CRD. This indicates that binding of K2 and L2 to galectin-3 induces changes of galectin-3 conformation including its CRD domain. The bigger galectin-3 conformation changes induced by K2 than L2 shown in CD is in line with the stronger binding to galectin-3 (Fig. 1) and stronger inhibition of galectin-3-activity (Fig. 3 and Fig S1) by K2 than L2. These suggest that galectin-3 conformation changes in response to binding of these compounds contribute to their inhibitory effect on galectin-3-mediated activities in cancer and immune cells.

### K2 inhibits tumour growth and metastasis in chick embryo model

Having revealed significant effects of the compounds on galectin-3-mediated cancer cell activities in vitro, K2 was selected and investigated on its effect on tumour growth and metastasis in chick embryos.

**Fig. 7:**
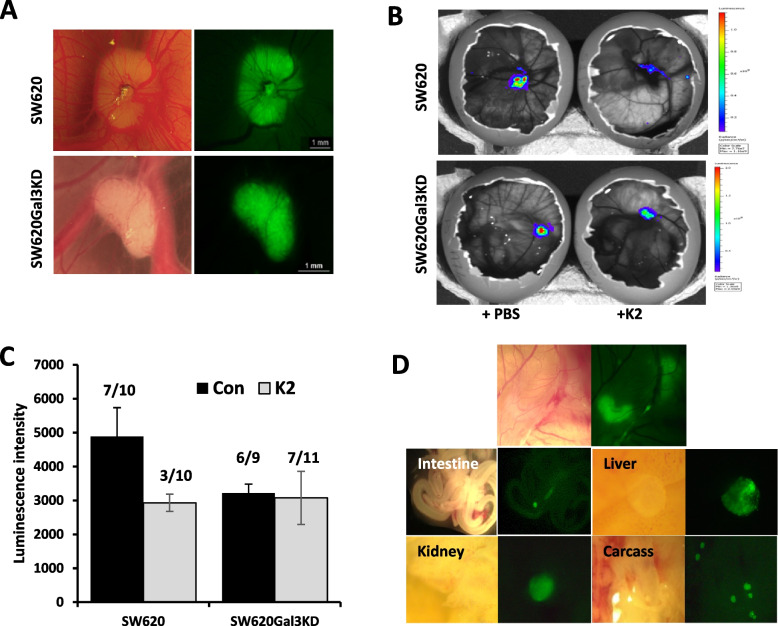
K2 inhibits tumour growth and metastasis in chicken embryos. Effect of K2 on tumour formation, growth and metastasis of SW620 and SW620Gal3KD was assessed in chick embryos. Tumour cells were engrafted to the chick chorioallantoic membrane (CAM) on embryonic day 7. K2 (24 µM) was injected into the allantoic cavity on the following day. Tumour formation and growth was analysed on embryonic day 14. Representative fluorescence (**A**) and IVIS (**B**) images of the tumours in the chick embryos are shown. Luminescence intensity of the tumours is shown in **C** (number of embryos formed tumour vs total embryos are shown on top of each group). Representative fluorescence images in different embryo organs grafted with SW620 cells are shown in **D**

When SW620 cells, without or with galectin-3 shRNA knockdown, were applied to the chick embryo model, 70% (7/10) and 67% (6/9), respectively, embryos formed tumours when applied with SW620 and galectin-3-knockdown SW620 cells (Fig. 7A-C). When K2 was injected to the allantoic cavity of embryos, the embryos that formed tumours of SW620 group reduced to 30% (3/10) while the embryos of the galectin-3 knockdown SW620 group was seen to be more than doubled (67%, 7/10). When the luminescence intensity of the tumours formed in the embryos was compared, K2 treated group showed 40% lower in comparison to the control PBS group (2931 vs 4886). On the other hand, little difference of the luminescence intensity was seen between K2 and PBS administration groups implanted with the galectin-3 knockdown SW620Gal3KD cells (3077 vs 3219). Further dissection of the organs of the embryos showed that 25% of the embryos with tumours showed metastasis to other organs such as liver, kidney, intestine, etc. (Fig. 7D) while none of the embryos with tumours and K2 administration showed metastasis in other organs. These results indicate that galectin-3 expression enhances tumour formation, growth and metastasis in the chicken embryo and K2 administration can largely prevent the galectin-3-mediated promotion of tumour growth and metastasis.

### K2 inhibit tumour growth and metastasis in mice

Having shown a potent effect of K2 on inhibition of galectin-3-mediated tumour growth and metastasis in the chick embryos, its effect on tumour growth and metastasis was further investigated in mice.

**Fig. 8:**
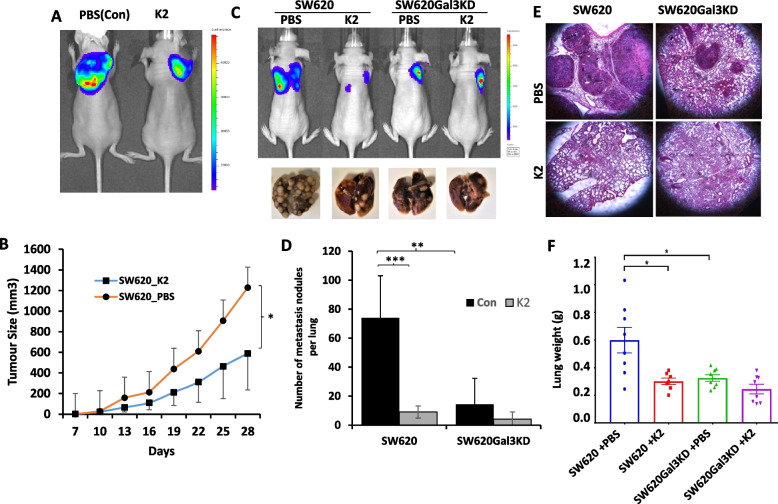
K2 inhibits tumour growth and metastasis in mice. In the tumour growth experiment (**A**, **B**), 18 Balb/c nude mice were grafted with 1 × 10^6^ luciferase-transfected SW620 cells subcutaneously. The mice were randomly divided into two equal groups and i.p. injected K2 (5 mg/kg) or PBS (control group), 3 times a week for 4 weeks. Tumour sizes were measured by clippers every three days (**B**) as well as weekly by IVIS. Panel A shows representative IVIS images of the mice after three weeks. In the metastasis experiment (**C**-**F**), 32 Balb/c nude mice were randomly divided into four equal groups. Two groups were intravenously injected with 2 × 10.^6^ SW620 cells and two with SW620Gal3KD cells via the animal tail vein. K2 (10 mg/kg) or PBS was *i.v* administrated daily for 5 consecutive days in the first week then once a week s.c. for seven weeks. Metastasis was monitored by IVIS imaging weekly. Representative IVIS images of the animals are shown in **C**. The animals were killed, and the lungs were excised. Representative lung images are shown at the bottom of **C**. The number of metastasis nodules on the surface of the lungs were quantified (**D**). Panel E shows representative images of H & E stained lung sections and F shows the lung weights of the different groups of the animals. *p < 0.05, ** *P* < 0.01, *** *P* < 0.001 (t tests)

For assessment on tumour growth, nude mice were inoculated subcutaneously with SW620 and then administrated K2 or PBS (control) intraperitoneally three-times a week for four weeks. All mice formed measurable tumours after one week. Tumour growth in the K2-treated group showed slower in comparison to the control group. At end of the fourth week, tumours in the K2 groups were significantly (51%) smaller than that in the control group (Fig. 8A, B). No difference of animal body weights was seen between the control and treated groups (Fig S3A).

To assess the effect of K2 on metastasis, SW620 and galectin-3 knockdown SW620Gal3KD cells were intravenously injected to the animal tail veins. The mice were then administrated by intravenous injection of K2 or PBS daily for five days then once a week for eight weeks. At end of the eight weeks, the animals were sacrificed and the number of metastasis nodules on the surface of the lungs were quantified. Significantly fewer metastasis nodules were observed in the group injected with SW620Gal3KD than with SW620 cells (Fig. 8C-E). Administration of K2 resulted in marked reduction (88%) of the number of metastasis nodules inoculated with SW620 in comparison to the PBS group. Similar results were observed in H & E stained lung sections. Administration of K2 on the other hand did not show significant effect on the number of metastasis nodules in mice inoculated with SW620Gal3KD. The lung weights in the K2 treated SW620 mice was also seen to be significantly lighter in comparison to the control group (Fig. 8F) while the animal body weights did not show significant differences between any groups (Fig S3B). Together, these results demonstrated the effectiveness of these novel galectin-3 inhibitors on inhibition of galectin-3-mediated tumour growth and metastasis in vivo.

## Discussion

This study reveals two non-carbohydrate small molecule compounds as potent galectin-3 inhibitors These closely related compound inhibitors, which share the same molecular compositions with difference of only the position of an NH2 group on an aromatic ring, bind to galectin-3 at low micromolar level. Molecular docking analysis suggests binding of the compounds on the canonical S-face of the galectin-3 CRD. These compound inhibitors were shown to inhibit galectin-3 binding to its ligands and inhibit galectin-3-mediated cancer cell adhesion, invasion, angiogenesis, as well as inhibit macrophage secretion of pre-inflammatory cytokines. Administration of K2 significantly reduced galectin-3 mediated tumour growth and metastasis in vivo in chick embryo and in mice. Neither of these compounds showed detectable cytotoxicity and genotoxicity. These non-carbohydrate compounds, therefore, represent a novel class of galectin-3 inhibitors and offer great potential to be developed as galectin-3-targeted therapeutic drugs for cancer treatment.

Galectin-3 is synthesized in the cytoplasm but is expressed both inside and outside cells. Newly synthesized galectin-3 can be transported into the cell nucleus, through interaction with either nuclear localization sequence (NLS)-containing proteins or nuclear import proteins such as importin-α or importin-β (Funasaka et al. 2014b). It can also be transported into the cell membrane and extracellular space via non-classical pathway. Cytoplasmic galectin-3 is an apoptosis inhibitor and nuclear galectin-3 is a potential mRNA splicing promoter (Newlaczyl and Yu 2011), both actions via protein–protein interactions. Extracellular galectin-3 is involved in a range of cell–cell and cell-environment interactions through, so far known, interaction of its CRD with galactose-terminated glycans.

With increased realization of galectin-3 multi-mode actions in promotion of cancer development, progression and metastasis, there has been an exponential level of recent interest in the development of galectin-3-targeted therapeutics. This interest was also strongly encouraged by the discovery that mice with galectin-3 knockout displayed no overt developmental or physiological abnormalities under standard laboratory conditions (Colnot et al. 1998), suggesting that inhibition of the galectin-3 actions with specific and non-cytotoxic inhibitors is unlikely to have significant adverse impact on normal cell function and homeostasis. The developments of galectin-3-targeted therapeutics have thus far focused exclusively on the galectin-3 carbohydrate binding sites. Several natural polysaccharides, glyco-peptides, and carbohydrate-compounds conjugates have been reported as galectin-3 inhibitors in the past few years and show to inhibit galectin-3-mediated actions in cancer (Marino et al. 2023; Duckworth et al. 2015; Ahmed and AlSadek 2015; Blanchard et al. 2014; Guha et al. 2013). Some of these inhibitors have shown promising results in early phase clinical trials either alone or in combination with chemotherapy or immune therapy (Marino et al. 2023; Aslanis et al. 2023; Curti et al. 2021; Keizman 2023). All of those galectin-3 inhibitors are, however, either wholly or partially carbohydrates and face the typical challenges associated with carbohydrate-based drugs due to poor physicochemical properties of carbohydrates (e.g. poor bioavailability, poor biodistribution and poor stability). Using a fluorescence-based screening platform [conjugation of galectin-3 with Europium and conjugation of TREM2 (Triggering receptor expressed on myeloid cells-2) with Alexa Fluor 647), a small molecule compound MG257 has been reported to be a galectin-3 binding inhibitor and was shown to inhibit galectin-3-induced TREM2 signalling in natural killer cells (Gabr et al. 2020). Several thiazole-linked coumarin piperazine compounds were also reported to be binding inhibitors to galectin-1 (Sethi et al. 2024). These non-carbohydrate galectin inhibitors will aid effective development of novel therapeutics against galectins including galectin-3.

As the sole chimera-type galectin, galectin-3 contains an ~ 130 amino acids carbohydrate-recognition domain (CRD) at its C-terminal which is Linked to a proline-rich, flexible N-terminal domain by a 10–15 amino acid linker region. The CRD domain is the main galectin-3 action site that interacts with galactose-terminated glycans. Its N-terminal domain facilitates galectin-3 formation of higher-order structures such as dimers and pentamers that contribute to extracellular galectin-3 actions (Johannes et al. 2022). It is fascinating that non-carbohydrate compounds K2 and L2 are shown to effectively inhibit galectin-3 binding and prevent galectin-3-mediated actions in cancer. Molecular docking analyses using state-of-the-art generative learning algorithms in programmes such as DynamicBind show that both K2 and L2 bind to the canonical carbohydrate binding site on the S-face of galectin-3 CRD. The important pi-cation interaction involving the non-conserved Arg144 with the aromatic anilino group are present in both the K2 and L2 complexes providing explanation as to why these compounds are able to bind to galectin-3. Furthermore, hydrophobic interactions involving Trp181 found in the analyses of the docked complexes are confirmed by a change in the Trp181 fluorescence. Interestingly, Trp181 was also suggested to be involved in the interaction of MG257 with galectin-3 (Gabr et al. 2020) which provides further confidence that the ligands are docked in the correct face of the protein. Since Arg144 is a non-conserved residue in galectins, a ligand interaction involving Arg144 will be a very desirable feature to confer binding specificity of ligands for galectin-3.

The observation of conformation changes in Circular Dichroism analysis of galectin-3 β-sheet, the key secondary structure of galectin-3 CRD, in response to the compound binding is in keeping with interaction of the compounds on galectin-3 CRD. Both compounds are driven by the same protein–ligand interactions; this is to be expected as the position of the amino group in the aniline makes little difference to the important interactions that mediate the binding between the compounds and galectin-3.

As a multi-functional molecule, galectin-3 promotes a range of steps in cancer development, progression, and metastasis (Marino et al. 2023), intracellularly and extracellularly (Girotti et al. 2020). The extracellular actions of galectin-3 are so far known to be exclusively mediated by binding of the galectin-3 CRD to galactose-terminated glycans. Many cell surface molecules and basement matrix proteins e.g. growth factor receptors, adhesion molecules and death receptors (Barrow et al. 2011a; Zhao et al. 2010; Kouo et al. 2015; Pang et al. 2022; Reticker-Flynn and Bhatia 2015), have been reported to carry galactose-terminated N- or O-linked glycans and are involved in galectin-3-mediated cancer promotion and immune cell activities. In this study, extracellular galectin-3-mediated tumour cell adhesion to matrix proteins, adhesion to and invasion through endothelial cells, angiogenesis and secretion of pro-inflammatory cytokines by macrophages, all of which are known to promote cancer progression, metastasis and immune invasion, are inhibitable by the presence of K2 and L2. This suggests that K2 and L2 can effectively inhibit galectin-3-mediated actions in cancer promotion. Indeed, administration of K2 is shown to significantly inhibit galectin-3-mediated tumour growth and metastasis in both chick embryo and mice models.

The chick chorioallantoic membrane (CAM) model has been used as a less expensive and complementary in vivo model to murine models in studying tumour development, angiogenesis and metastasis (Deryugina and Quigley 2008). This highly reproductive system is a simple way to study complex biological processes, such as angiogenesis, with well-developed, transparent and highly vascularised tissue, in vivo. The chorioallantoic membrane is highly vascularised and efficiently supports the growth of inoculated tumour cells. The dense capillary network of the membrane also serves as an escape route for aggressive cells in their invasion into the chick’s vasculature and internal organs to form micro metastatic foci in a short time frame. CAM is increasingly used as a complementary pre-clinical model in studying cancer therapeutics (Kue et al. 2015). When K2 was tested in this model with inoculation of human colon cancer SW620 cells, clear inhibition of tumour growth and metastasis was observed while no effect of K2 was observed in the embryos inoculated with galectin-3-knockdown SW620Gal3KD cells.

The effectiveness of these galectin-3 inhibitors on tumour growth and metastasis were further demonstrated in mice. When K2 was administrated three times a week for four weeks, the size of tumour inoculated with SW620 cells was reduced by ~ 50% in comparison to that in the control mice. Lung metastasis inoculated with SW620 cells further showed to be reduced by more than 80% when K2 was administrated, while no obvious effect of K2 was observed in the mice inoculated with galectin-3 knockdown SW620Gal3KD cells. These results indicate that these compounds inhibitors can effectively prevent galectin-3-mediated promotion of tumour growth and metastasis in vivo. This, together with the observation of their lack of cytotoxicity and genotoxicity, suggests that these non-carbohydrate small molecule galectin-3 inhibitors represent a novel class of galectin-3 inhibitors and can offer significant potential to be developed as galectin-3-targeted novel therapeutic drugs for cancer treatment.

It should be emphasised that galectin-3 is not only involved in cancer pathogenesis but also in the pathogenesis of several other diseases such as fibrosis (Bouffette et al. 2023; Blanda et al. 2020; Boer et al. 2009), inflammation (Bouffette et al. 2023) and diabetes (Seferovic et al. 2014). Galectin-3 does so, again, through multiple molecular mechanisms by interaction mostly with various galactose-terminated glycans on cell surface molecules. For example, galectin-3 promotes fibrogenesis by binding to integrins on fibroblast cells and activating the profibrotic mediator transforming growth factor-β1 (TGF-β1) (Calver et al. 2024). Interaction of galectin-3 with macrophages increases secretion of pro-inflammatory cytokines such as IL-6 and TNFα that promotes inflammation and tissue fibrosis (Li et al. 2014). Interfering these galectibn-3 actions is believed to have implication for therapeutic treatment of those disease. Indeed, several polysaccharides and carbohydrate-compound conjugates of galectin-3 inhibitors have demonstrated encouraging results in early phase clinical trials in those disease areas (Aslanis et al. 2023; Hirani 2021; Chalasani et al. 2020; Harrison et al. 2016). The discovery of a novel class of non-carbohydrate galectin-3 inhibitors without cytotoxicity shown in this study therefore not only offers great opportunities for therapeutic treatment of cancer but also of those diseases that are also in high unmet clinical needs.

It should be mentioned that although galectin-3 is involved in the maintenance of homeostasis, it has been reported previously that mice with knockout galectin-3 displayed no overt developmental or physiological abnormalities under standard laboratory conditions (Colnot et al., 1998, ref 1x). Inhibition of the galectin-3 actions with specific and non-cytotoxic inhibitors is therefore unlikely to have significant impact on normal cell function and homeostasis.

Thus, non-carbohydrate small molecule compounds K2 and L2 are potent galectin-3 inhibitors that can effectively inhibit galectin-3-mediated actions in cancer progression and metastasis both in vitro and in vivo. These novel, non-carbohydrate galectin-3 inhibitor compounds offer great promise and potential to be developed as galectin-3 targeted therapeutic drugs for cancer treatment. As galectin-3 is known to also promote the pathogenesis of inflammation and fibrosis-associated diseases (Ahmed et al. 2023) and is being pursued as a therapeutic target in those diseases (Bouffette et al. 2023), the revelation of those non-carbohydrate compound inhibitors also opens new avenues of the development of galectin-3-targeted therapeutics in those disease areas.

## Supplementary Information

Supplementary Material 1

## Data Availability

No datasets were generated or analysed during the current study.

## Abbreviations

ASF: Asialo-fetuin
CAM: Chick chorioallantoic membrane
CD: Circular Dichroism
DMEM: Dulbecco’s modified Eagle medium
CRD: Carbohydrate-recognition domain
Gal3: Galectin-3
Gal3KD: Galectin-3 knockdown
Gal3KO: Galectin-3 Knockout
HUVECs: Human umbilical vein endothelial cells
LAR: Luciferase Assay Reagent
LDH: Lactate dehydrogenase
PLB: Passive Lysis Buffer
TEER: Trans-endothelial electric resistance
TFS: Tryptophan Fluorescence Spectroscopy
