# Supplementary material for: Targeting galectin-3 in cancer by novel and unique inhibitors of non-carbohydrate origin
Source: Mol Med. 2025 Sep 29;31:299. doi: 10.1186/s10020-025-01356-6 (PMC12482042; doi:10.1186/s10020-025-01356-6)
Supplement: Supplementary file 1 — Supplementary Material 1 [file 10020_2025_1356_MOESM1_ESM.pptx]

## Slide 1
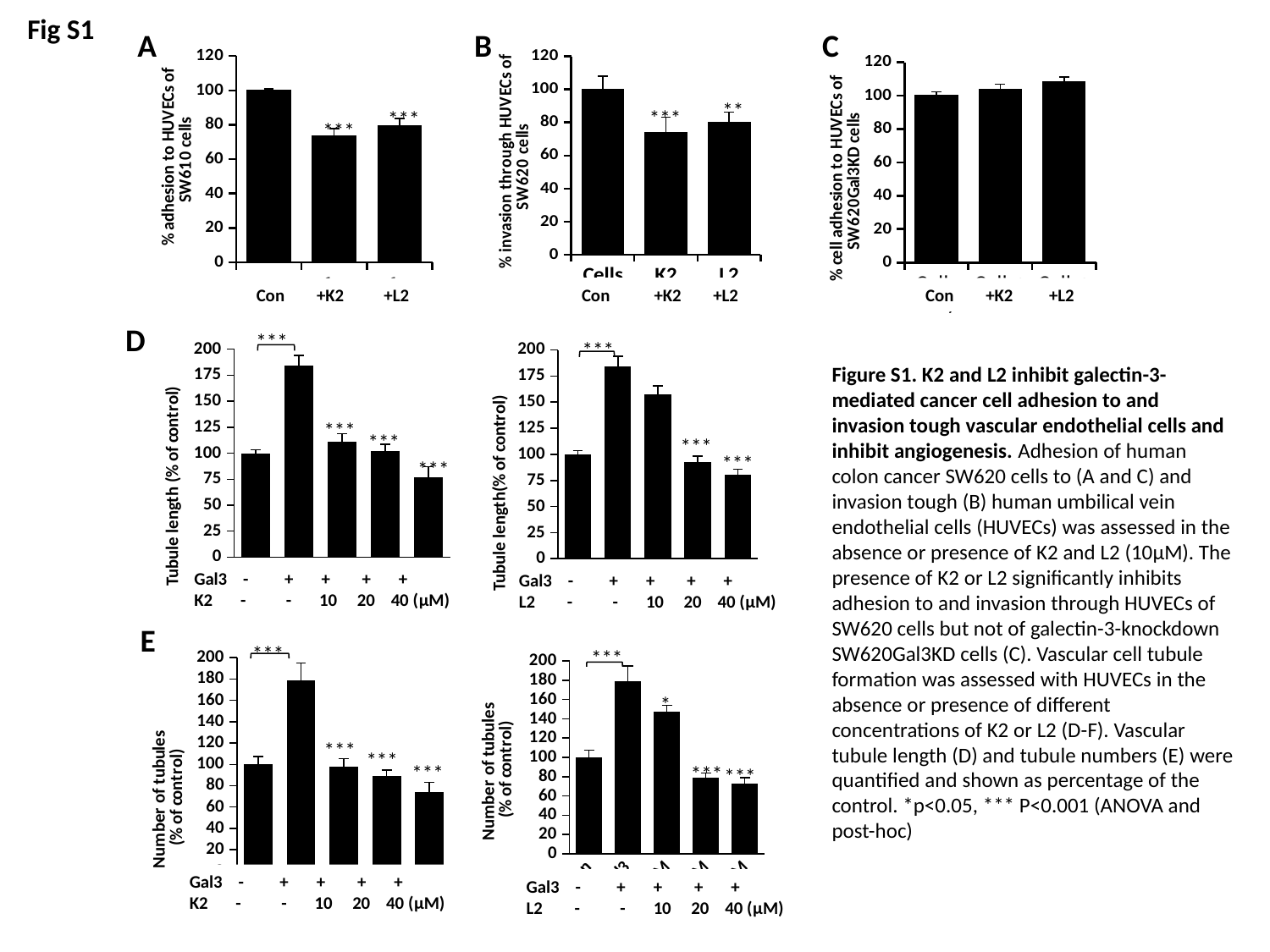

Fig S1
A
B
C
### Chart
| Category | |
|---|---|
| Cells Only | 100.00019322600292 |
| Cells+K2 | 73.4678330627292 |
| Cells+L2 | 79.5450765789006 |***
 Con +K2 +L2
***
### Chart
| Category | |
|---|---|
| Cells Only | 100.00208833663986 |
| K2 | 73.95217709094706 |
| L2 | 80.32369217917929 |**
***
 Con +K2 +L2
### Chart
| Category | |
|---|---|
| Cells Only | 100.0 |
| Cells+K2 | 103.57757283834913 |
| Cells+L2 | 108.16730030347014 | Con +K2 +L2
D
***
***
***
***
### Chart
| Category | |
|---|---|
| Con | 100.00001495252761 |
| Gal3 | 184.32300749159015 |
| Gal3+10µM | 111.21755067878867 |
| Gal3+20µM | 102.0404181773392 |
| Gal3+40µM | 77.12261035245723 |***
***
***
### Chart
| Category | |
|---|---|
| Con | 100.00001495252761 |
| Gal3 | 184.32300749159015 |
| Gal3+10µML2 | 157.31726983293913 |
| Gal3+20µML2 | 92.6769883964648 |
| Gal3+40µML2 | 80.74931975344776 |Figure S1. K2 and L2 inhibit galectin-3-mediated cancer cell adhesion to and invasion tough vascular endothelial cells and inhibit angiogenesis. Adhesion of human colon cancer SW620 cells to (A and C) and invasion tough (B) human umbilical vein endothelial cells (HUVECs) was assessed in the absence or presence of K2 and L2 (10µM). The presence of K2 or L2 significantly inhibits adhesion to and invasion through HUVECs of SW620 cells but not of galectin-3-knockdown SW620Gal3KD cells (C). Vascular cell tubule formation was assessed with HUVECs in the absence or presence of different concentrations of K2 or L2 (D-F). Vascular tubule length (D) and tubule numbers (E) were quantified and shown as percentage of the control. *p<0.05, *** P<0.001 (ANOVA and post-hoc)
Gal3 - + + + +
K2 - - 10 20 40 (µM)
Gal3 - + + + +
L2 - - 10 20 40 (µM)
E
***
***
***
***
### Chart
| Category | |
|---|---|
| Con | 100.0 |
| Gal3 | 178.9272030651341 |
| Gal3+10uM | 97.70114942528735 |
| Gal3+20µM | 89.272030651341 |
| Gal3+40 µM | 73.9463601532567 |***
*
***
***
### Chart
| Category | |
|---|---|
| Con | 100.0 |
| Gal3 | 178.9272030651341 |
| Gal3+10uM | 147.5095785440613 |
| Gal3+20µM | 79.3103448275862 |
| Gal-3+40 µM | 73.1800766283525 |Gal3 - + + + +
K2 - - 10 20 40 (µM)
Gal3 - + + + +
L2 - - 10 20 40 (µM)

## Slide 2
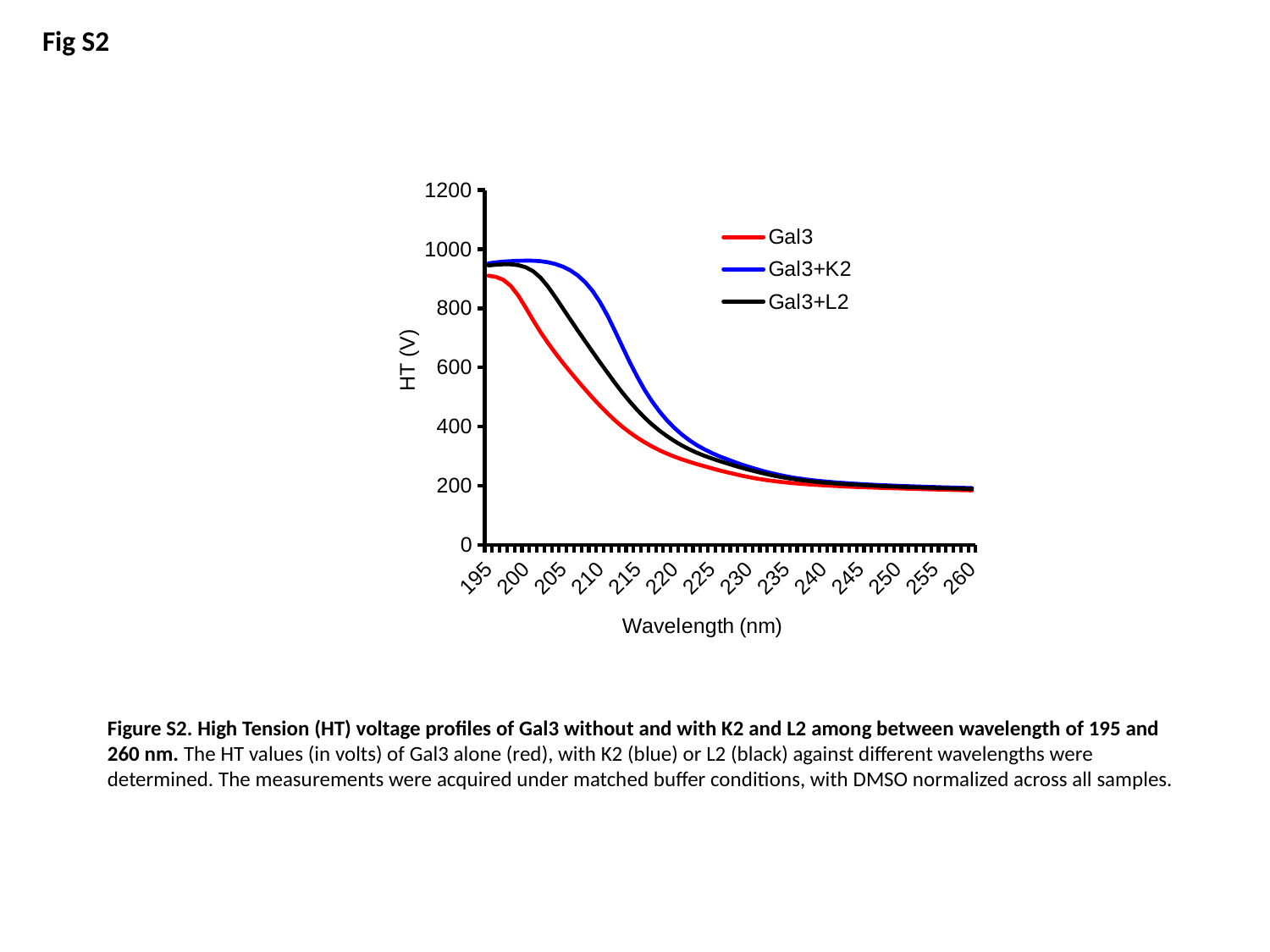

Fig S2
### Chart
| Category | Gal3 | Gal3+K2 | Gal3+L2 |
|---|---|---|---|
| 260 | 184.736 | 192.459 | 189.567 |
| 259 | 185.328 | 193.153 | 190.273 |
| 258 | 185.876 | 193.882 | 190.986 |
| 257 | 186.571 | 194.467 | 191.704 |
| 256 | 187.392 | 195.208 | 192.413 |
| 255 | 187.937 | 195.949 | 193.104 |
| 254 | 188.65 | 196.645 | 193.78 |
| 253 | 189.302 | 197.503 | 194.543 |
| 252 | 189.852 | 198.335 | 195.382 |
| 251 | 190.698 | 199.391 | 196.216 |
| 250 | 191.599 | 200.262 | 197.139 |
| 249 | 192.21 | 201.266 | 198.039 |
| 248 | 193.029 | 202.331 | 199.163 |
| 247 | 194.045 | 203.425 | 200.331 |
| 246 | 194.843 | 204.638 | 201.509 |
| 245 | 195.802 | 205.992 | 202.921 |
| 244 | 196.745 | 207.592 | 204.311 |
| 243 | 197.903 | 209.263 | 205.733 |
| 242 | 199.133 | 211.047 | 207.422 |
| 241 | 200.363 | 212.998 | 209.405 |
| 240 | 201.878 | 215.189 | 211.44 |
| 239 | 203.341 | 217.801 | 213.817 |
| 238 | 205.098 | 220.604 | 216.509 |
| 237 | 206.918 | 224.139 | 219.517 |
| 236 | 209.068 | 227.831 | 222.98 |
| 235 | 211.478 | 232.336 | 226.829 |
| 234 | 214.248 | 237.487 | 231.26 |
| 233 | 217.294 | 243.29 | 236.369 |
| 232 | 220.921 | 249.795 | 242.037 |
| 231 | 224.901 | 256.796 | 248.197 |
| 230 | 229.683 | 264.375 | 254.674 |
| 229 | 234.888 | 272.408 | 261.336 |
| 228 | 240.517 | 281.37 | 268.774 |
| 227 | 246.551 | 290.725 | 276.576 |
| 226 | 252.651 | 300.421 | 284.406 |
| 225 | 259.519 | 311.537 | 292.888 |
| 224 | 266.614 | 323.907 | 301.906 |
| 223 | 273.721 | 337.822 | 312.098 |
| 222 | 281.38 | 354.43 | 323.641 |
| 221 | 289.762 | 373.618 | 336.667 |
| 220 | 298.751 | 395.755 | 351.436 |
| 219 | 308.787 | 421.26 | 368.133 |
| 218 | 320.168 | 451.114 | 386.576 |
| 217 | 332.931 | 485.259 | 407.215 |
| 216 | 347.147 | 524.25 | 430.44 |
| 215 | 362.916 | 568.91 | 456.381 |
| 214 | 380.431 | 617.98 | 484.791 |
| 213 | 399.708 | 670.738 | 515.587 |
| 212 | 421.526 | 724.302 | 548.541 |
| 211 | 445.095 | 775.595 | 582.828 |
| 210 | 470.631 | 820.88 | 617.409 |
| 209 | 497.893 | 858.976 | 653.088 |
| 208 | 526.087 | 888.689 | 689.2 |
| 207 | 555.323 | 911.584 | 725.505 |
| 206 | 585.186 | 928.577 | 762.573 |
| 205 | 616.129 | 940.987 | 800.241 |
| 204 | 648.589 | 950.031 | 837.973 |
| 203 | 683.014 | 955.875 | 873.831 |
| 202 | 719.918 | 959.515 | 903.967 |
| 201 | 760.068 | 961.181 | 925.583 |
| 200 | 802.971 | 961.19 | 939.086 |
| 199 | 843.63 | 960.774 | 946.02 |
| 198 | 875.983 | 959.497 | 948.686 |
| 197 | 896.536 | 957.974 | 948.901 |
| 196 | 906.346 | 955.492 | 947.571 |
| 195 | 910.594 | 952.145 | 944.992 |Figure S2. High Tension (HT) voltage profiles of Gal3 without and with K2 and L2 among between wavelength of 195 and 260 nm. The HT values (in volts) of Gal3 alone (red), with K2 (blue) or L2 (black) against different wavelengths were determined. The measurements were acquired under matched buffer conditions, with DMSO normalized across all samples.

## Slide 3
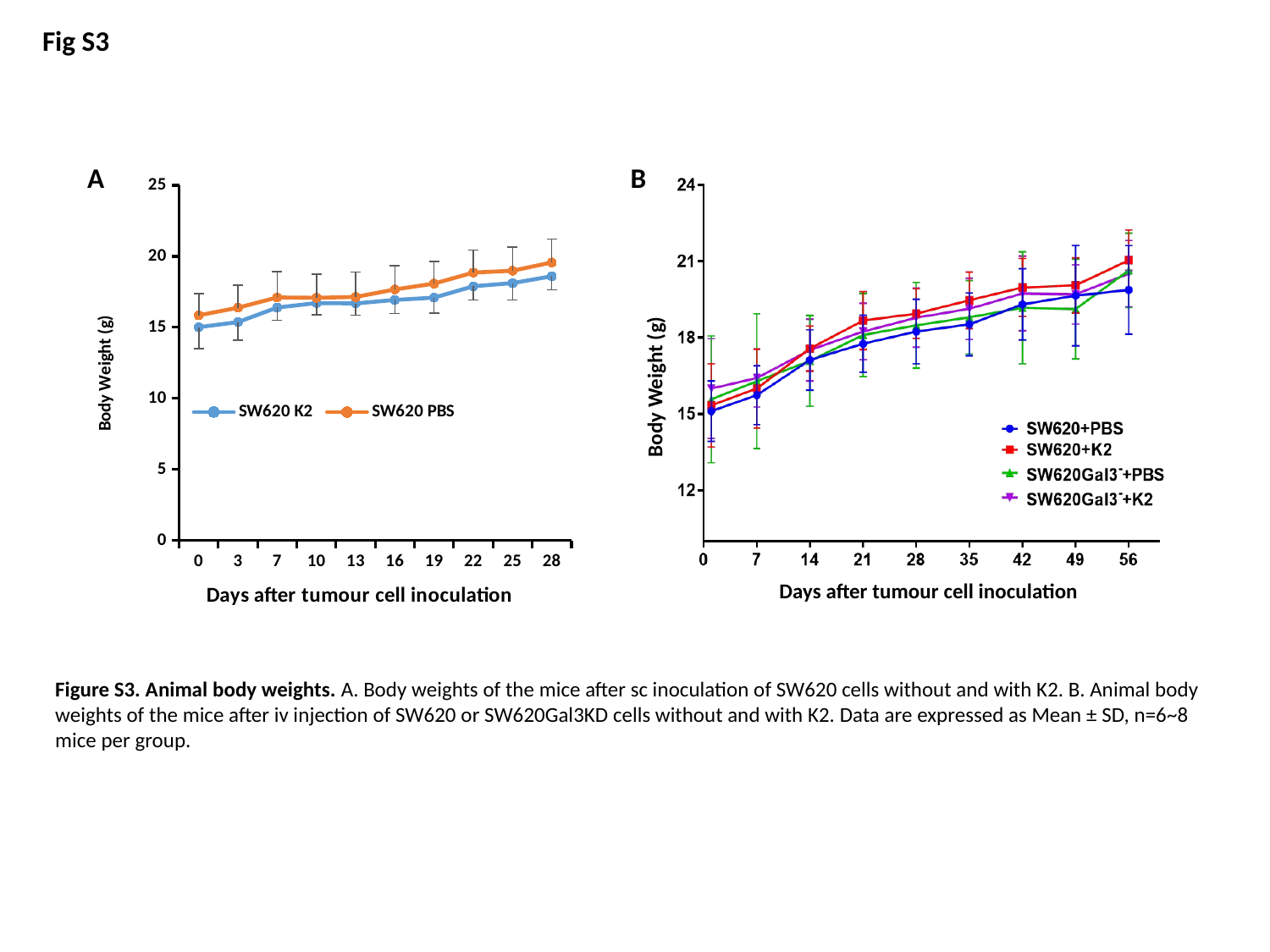

Fig S3
B
A
### Chart
| Category | SW620 K2 | SW620 PBS |
|---|---|---|
| 0 | 15.016666666666667 | 15.85 |
| 3 | 15.373333333333333 | 16.385714285714286 |
| 7 | 16.396666666666665 | 17.10857142857143 |
| 10 | 16.715 | 17.082857142857144 |
| 13 | 16.686666666666667 | 17.141428571428573 |
| 16 | 16.933333333333334 | 17.67142857142857 |
| 19 | 17.093333333333334 | 18.081428571428575 |
| 22 | 17.895 | 18.859999999999996 |
| 25 | 18.116666666666664 | 18.985714285714288 |
| 28 | 18.599999999999998 | 19.571428571428573 |Body Weight (g)
Days after tumour cell inoculation
Figure S3. Animal body weights. A. Body weights of the mice after sc inoculation of SW620 cells without and with K2. B. Animal body weights of the mice after iv injection of SW620 or SW620Gal3KD cells without and with K2. Data are expressed as Mean ± SD, n=6~8 mice per group.

## Slide 4
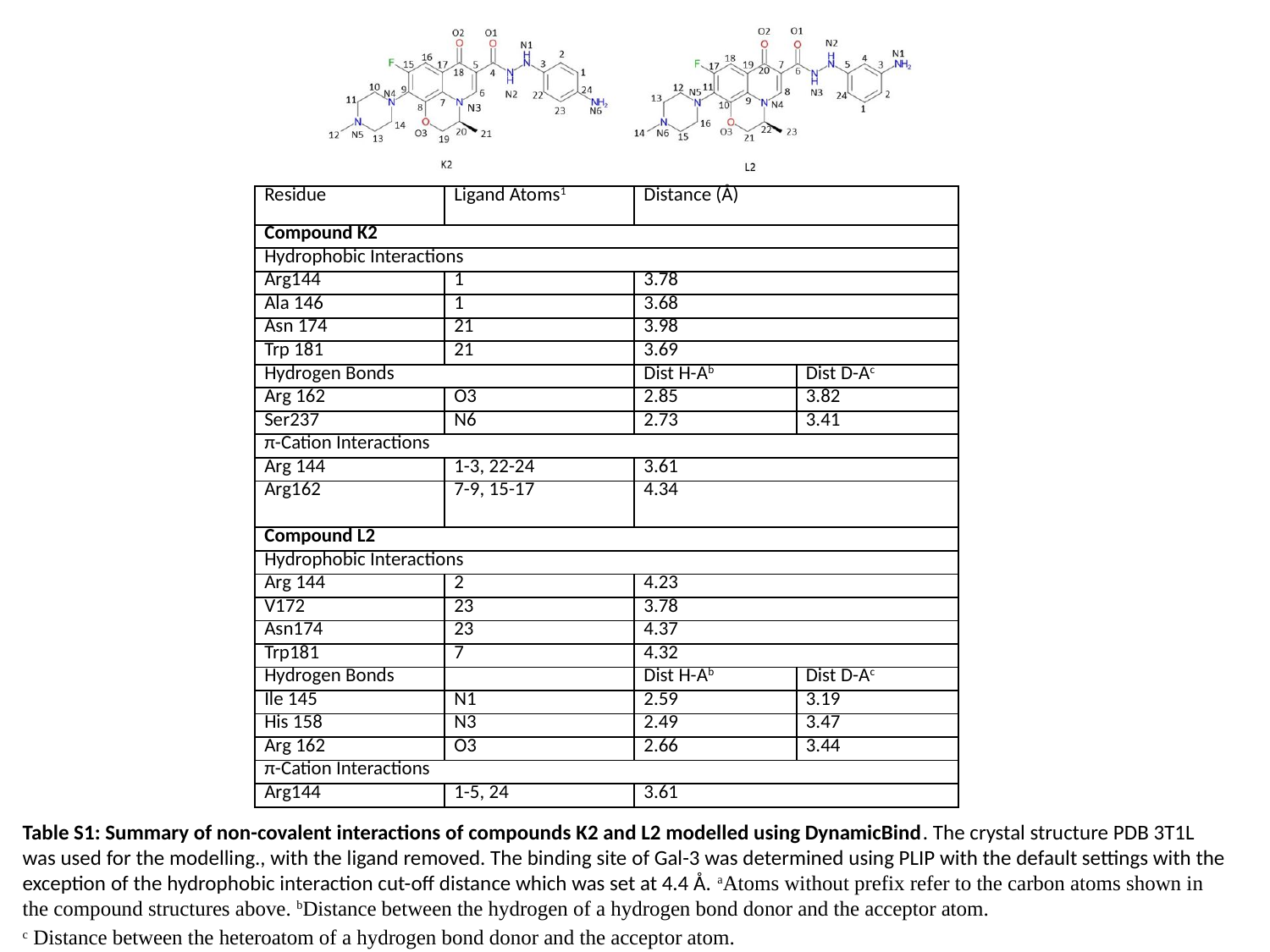

| Residue | Ligand Atoms1 | Distance (Å) | |
| --- | --- | --- | --- |
| Compound K2 | | | |
| Hydrophobic Interactions | | | |
| Arg144 | 1 | 3.78 | |
| Ala 146 | 1 | 3.68 | |
| Asn 174 | 21 | 3.98 | |
| Trp 181 | 21 | 3.69 | |
| Hydrogen Bonds | | Dist H-Ab | Dist D-Ac |
| Arg 162 | O3 | 2.85 | 3.82 |
| Ser237 | N6 | 2.73 | 3.41 |
| π-Cation Interactions | | | |
| Arg 144 | 1-3, 22-24 | 3.61 | |
| Arg162 | 7-9, 15-17 | 4.34 | |
| Compound L2 | | | |
| Hydrophobic Interactions | | | |
| Arg 144 | 2 | 4.23 | |
| V172 | 23 | 3.78 | |
| Asn174 | 23 | 4.37 | |
| Trp181 | 7 | 4.32 | |
| Hydrogen Bonds | | Dist H-Ab | Dist D-Ac |
| Ile 145 | N1 | 2.59 | 3.19 |
| His 158 | N3 | 2.49 | 3.47 |
| Arg 162 | O3 | 2.66 | 3.44 |
| π-Cation Interactions | | | |
| Arg144 | 1-5, 24 | 3.61 | |
Table S1: Summary of non-covalent interactions of compounds K2 and L2 modelled using DynamicBind. The crystal structure PDB 3T1L was used for the modelling., with the ligand removed. The binding site of Gal-3 was determined using PLIP with the default settings with the exception of the hydrophobic interaction cut-off distance which was set at 4.4 Å. aAtoms without prefix refer to the carbon atoms shown in the compound structures above. bDistance between the hydrogen of a hydrogen bond donor and the acceptor atom.
c Distance between the heteroatom of a hydrogen bond donor and the acceptor atom.

## Slide 5
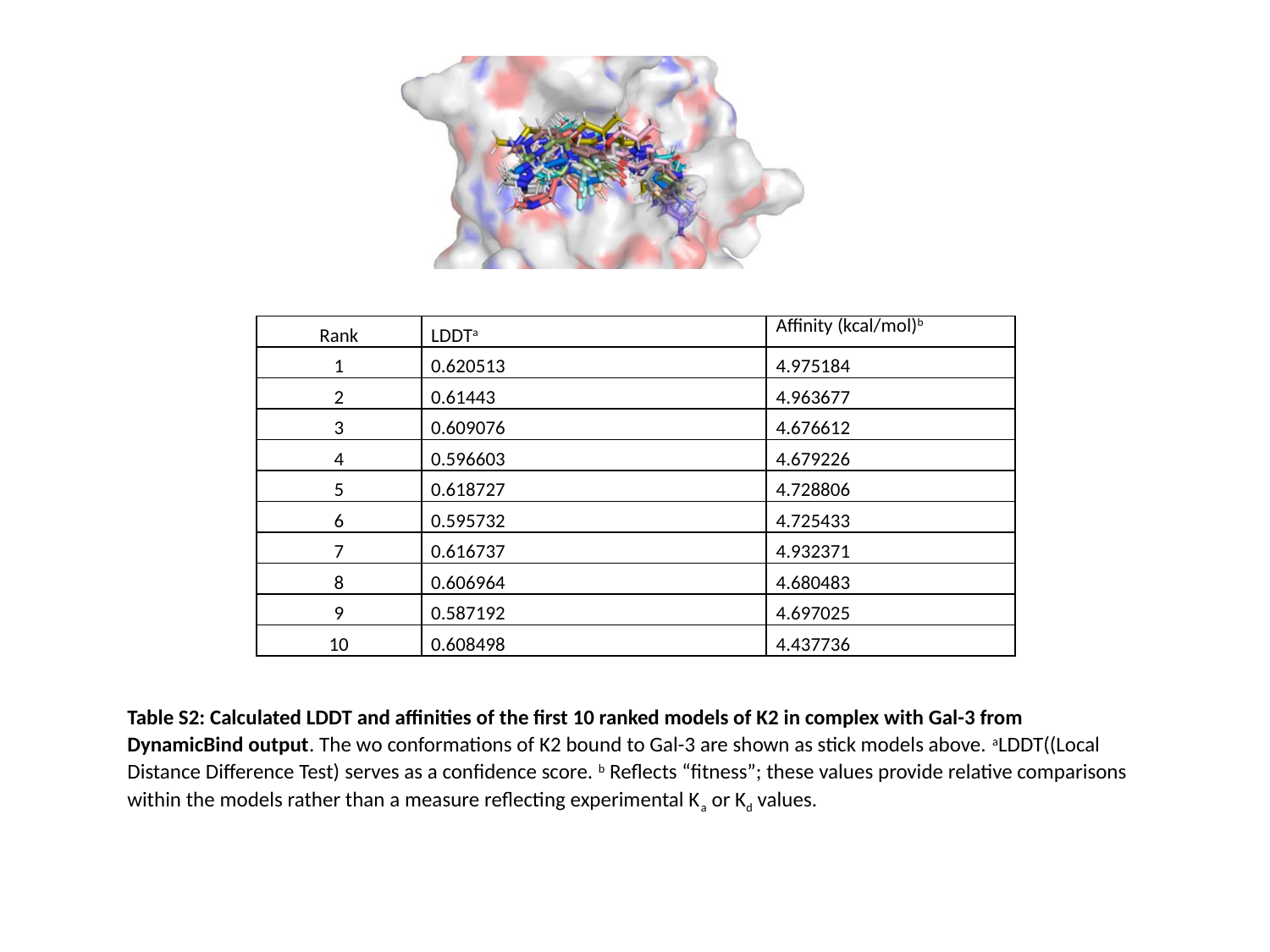

| Rank | LDDTa | Affinity (kcal/mol)b |
| --- | --- | --- |
| 1 | 0.620513 | 4.975184 |
| 2 | 0.61443 | 4.963677 |
| 3 | 0.609076 | 4.676612 |
| 4 | 0.596603 | 4.679226 |
| 5 | 0.618727 | 4.728806 |
| 6 | 0.595732 | 4.725433 |
| 7 | 0.616737 | 4.932371 |
| 8 | 0.606964 | 4.680483 |
| 9 | 0.587192 | 4.697025 |
| 10 | 0.608498 | 4.437736 |
Table S2: Calculated LDDT and affinities of the first 10 ranked models of K2 in complex with Gal-3 from DynamicBind output. The wo conformations of K2 bound to Gal-3 are shown as stick models above. aLDDT((Local Distance Difference Test) serves as a confidence score. b Reflects “fitness”; these values provide relative comparisons within the models rather than a measure reflecting experimental Ka or Kd values.

## Slide 6
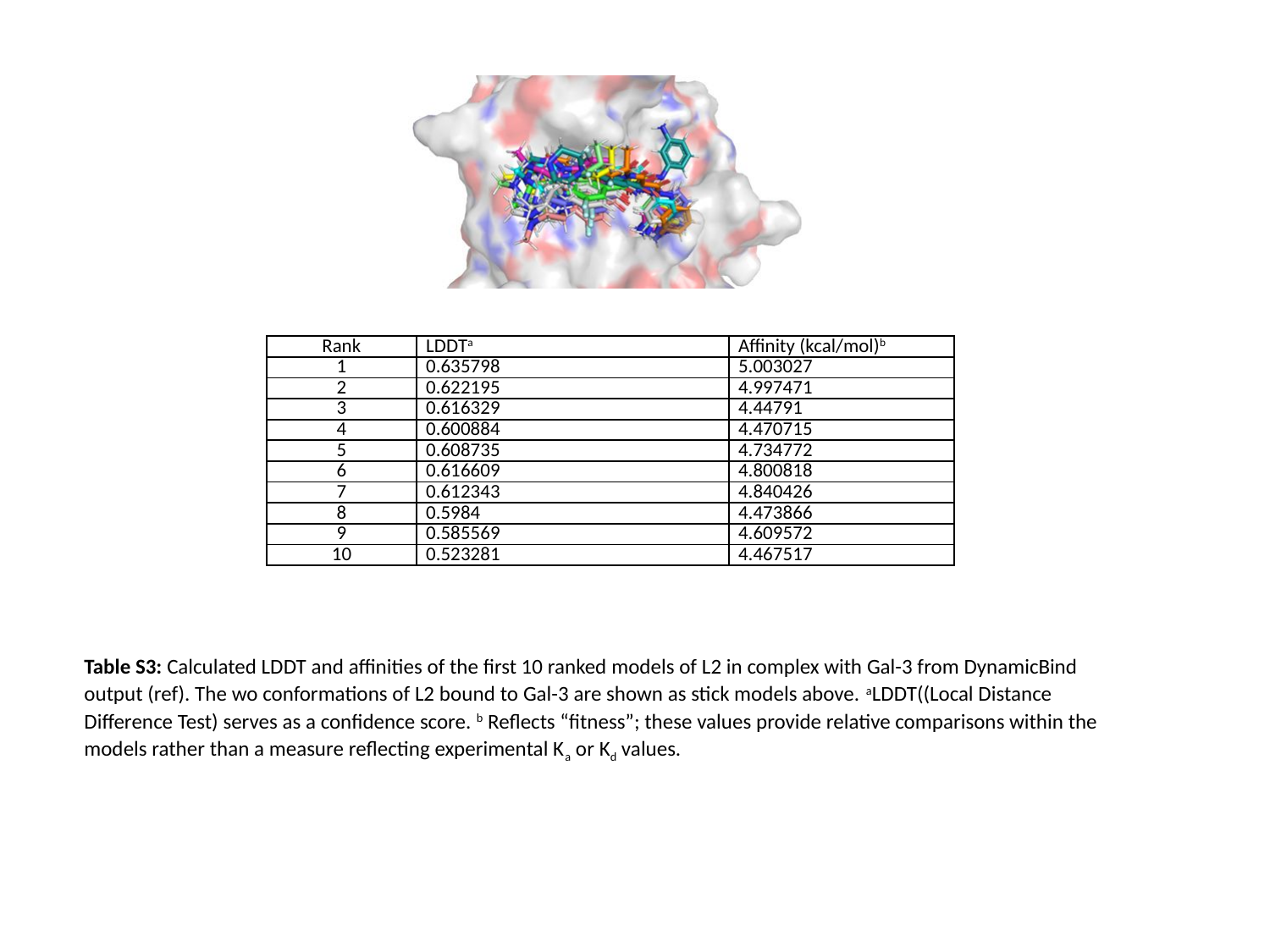

| Rank | LDDTa | Affinity (kcal/mol)b |
| --- | --- | --- |
| 1 | 0.635798 | 5.003027 |
| 2 | 0.622195 | 4.997471 |
| 3 | 0.616329 | 4.44791 |
| 4 | 0.600884 | 4.470715 |
| 5 | 0.608735 | 4.734772 |
| 6 | 0.616609 | 4.800818 |
| 7 | 0.612343 | 4.840426 |
| 8 | 0.5984 | 4.473866 |
| 9 | 0.585569 | 4.609572 |
| 10 | 0.523281 | 4.467517 |
Table S3: Calculated LDDT and affinities of the first 10 ranked models of L2 in complex with Gal-3 from DynamicBind output (ref). The wo conformations of L2 bound to Gal-3 are shown as stick models above. aLDDT((Local Distance Difference Test) serves as a confidence score. b Reflects “fitness”; these values provide relative comparisons within the models rather than a measure reflecting experimental Ka or Kd values.
